# Sex differences in colonization of gut microbiota from a man with short-term vegetarian and inulin-supplemented diet in germ-free mice

**DOI:** 10.1038/srep36137

**Published:** 2016-10-31

**Authors:** Jing-jing Wang, Jing Wang, Xiao-yan Pang, Li-ping Zhao, Ling Tian, Xing-peng Wang

**Affiliations:** 1 Shanghai Key Laboratory for Pancreatic Diseases, Shanghai General Hospital, Shanghai Jiao Tong University School of Medicine, Shanghai 201620, PR China; 2 State Key Laboratory of Microbial Metabolism, School of Life Sciences and Biotechnology, Shanghai Jiao Tong University, Shanghai 200240, PR China

## Abstract

Gnotobiotic mouse model is generally used to evaluate the efficacy of gut microbiota. Sex differences of gut microbiota are acknowledged, yet the effect of recipient’s gender on the bacterial colonization remains unclear. Here we inoculated male and female germ-free C57BL/6J mice with fecal bacteria from a man with short-term vegetarian and inulin-supplemented diet. We sequenced bacterial 16S rRNA genes V3-V4 region from donor’s feces and recipient’s colonic content. Shannon diversity index showed female recipients have higher bacteria diversity than males. Weighted UniFrac principal coordinates analysis revealed the overall structures of male recipient’s gut microbiota were significantly separated from those of females, and closer to the donor. Redundancy analysis identified 46 operational taxonomic units (OTUs) differed between the sexes. The relative abundance of 13 OTUs were higher in males, such as *Parabacteroides distasonis* and *Blautia faecis*, while 33 OTUs were overrepresented in females, including *Clostridium* groups and *Escherichia fergusonii/Shigella sonnei*. Moreover, the interactions of these differential OTUs were sexually distinct. These findings demonstrated that the intestine of male and female mice preferred to accommodate microbiota differently. Therefore, it is necessary to designate the gender of gnotobiotic mice for complete evaluation of modulatory effects of gut microbiota from human feces upon diseases.

The human gastrointestinal tract carries up to 10^14^ bacteria more than ten-fold the number of human cells in the body, which is causally related to human health via yielding metabolites, and/or interacting with the immune systems^1^,^2^. In the last decade, germ-free mouse model has been widely applied to demonstrate gut microbiota as a contributing factor in some human diseases^3^,^4^,^5^. As known, germ-free mice were resistant to high-fat and high-sugar diet-induced obesity^3^, and when they were transplanted the gut microbiota from the obese mice or human, they became adiposity^4^,^6^. On the contrary, transfer of gut microbiota from lean donors improved insulin sensitivity in individuals with metabolic syndrome^7^. That is to say that transplantation of the intact gut microbiota to germ-free mice is a good tool to study the function of the gut microbiota in the development and treatment of gut microbiota-related diseases.

However, gut microbiota showed sex bias in either human beings or animals^5^,^8^,^9^,^10^,^11^,^12^. It was identified that Bacteroidetes showed higher abundance in men than women according to an American study^8^. A cross-sectional study in European countries also showed that *Bacteroides-Prevotella* group was more prominent in male than female^11^. *Bacteroides thetaiotaomicron* was also identified as being higher in men than women in Chinese population^10^. Moreover, male mice held lower bacterial diversity^12^, and higher *Parabacteroides* belonging to Bacteroidetes than females, consistent with the above results of clinical trial^5^.

Intriguingly, the compositional and metabolic changes of gut microbiota response to diet, exogenous bacteria or genetic deletion also demonstrated sexual dimorphism^13^,^14^,^15^,^16^. Whether in wild and laboratory fish, laboratory mice or in human beings, the diet made different effects on gut microbiota composition between sexes^13^. An oligofructose-supplemented diet increased the abundance of Bacteroidetes in female rats, yet it did not change fecal community structure even if the butyrate levels increased in males^14^. Butyrate has been shown to attenuate gut inflammation by multiple mechanisms^17^,^18^,^19^,^20^,^21^. Probiotics also exerted dissimilar responses on female and male mice. After probiotic consumption, two genera *Staphylococcus* and *Roseburia* in Firmicutes were overrepresented in females, while butyrate and acetate were increased in males^15^. Deletion of Bmal1, a gene encoding a core molecular clock component, induced changes in the microbial composition based on sexes. In male mice, it decreased Proteobacteria (including *Helicobacter* and *Sutterella*) and increased TM7 (including F16 spp.); while in female mice, Cyanobacteria and Tenericutes were increased, but Proteobacteria and TM7 were unchanged^16^.

More importantly, sex differences in the gut microbiota play a key role in gender-related diseases^5^,^12^. Recently, it was found that the gut microbiome from specific pathogen-free (SPF) nonobese diabetic (NOD) male mice could protect the immature recipients of female from type 1 diabetes (T1D) by altering microbiota, elevating testosterone, and reducing islet inflammation and autoantibody production^5^.

Hence, sex is an important determinant to gut microbiota, and involves in the development of disease. However, how sex influence human gut microbiota colonization in germ-free mice has not been characterized so far. We wonder whether an intact human gut microbiota colonizes differently between male and female recipient mice, and what the differences are. Herein, we inoculated fecal suspension of a healthy man with short-term vegetarian and inulin-supplemented diet to male and female germ-free mice. By using high-throughput sequencing and bioinformatics analysis, we showed the colonization of gut microbiota was different in diversity, overall structure, and individual phylotype, and explored the interactions among differential bacteria between male and female recipient mice. These results provided a direct evidence for sexual dimorphism of gut microbiota colonization, which would suggest that both sexes of gnotobiotic mice should be applied to investigate the effects of gut microbiota.

## Results

### Alpha-diversity analysis of gut microbiota of male and female recipient

**Figure 1:**
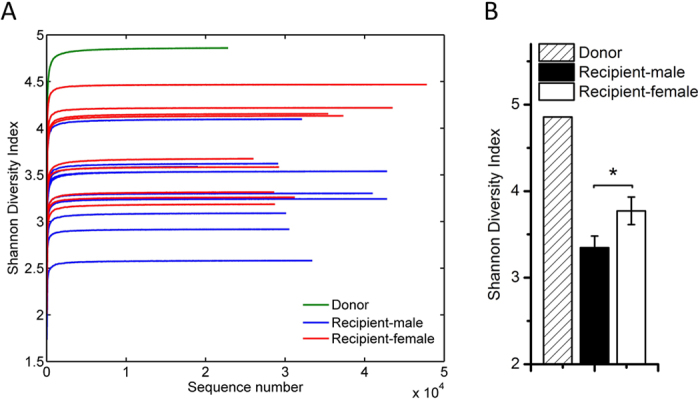
The colonization of gut microbiota in female recipient have higher alpha-diversity than that in male recipient. (**A**) Shannon Diversity Index curves; (**B**) Shannon diversity index of each group. Data were shown as means ± SEM. Differences were assessed by Mann-Whitney test. *P < 0.05. n = 1 for Donor; n = 10 for Recipient-male group; n = 9 for Recipient-female group.

To investigate whether gut microbiota derived from the same donor colonize in the recipients’ gut in a sex-dependent manner, we performed bacterial 16S rRNA gene V3-V4 region sequencing for the donor, 10 male and 9 female recipient’s colonic content samples on the Illumina MiSeq platform. A total of 698,564 usable high-quality raw reads were obtained. The sequences were binned into 2934 OTUs at the 97% similarity level. After chimeras and singleton removing, there remained 554 OTUs. Among those, 200, 148 ± 31 ((74 ± 5)% of the total OTUs in the donor), and 154 ± 38 ((77 ± 5)% of the total OTUs in the donor) OTUs existed in the gut of the donor, male and female recipients, respectively (Supplementary Figure S1A,B). There were 134 OTUs shared between the donor and the recipients, and 66 and 354 OTUs were unique in the donor and recipients, respectively. Theoretically, all the OTUs in recipient should come from the donor. The unique OTUs found in recipients might be dormant species in the donor’s gut that below the detection limit, and then become dominant after transplanted into mice. Shannon diversity indices were significantly higher in female recipient than male one (Fig. 1A,B), which suggested that more bacteria colonized in the female mice after inoculated with fecal microbiota of the donor. Observed Species curves showed that there was an increase trend in female mice than male mice, but the difference did not reach statistical significance (Supplementary Figure S2A,B).

### Taxonomy-based comparisons of gut microbiota at the phylum, family and genus levels

Taxonomic assignment showed that there were 4 phyla, 28 families and 58 genera in the feces of the donor and recipient (Supplementary Figure S3). The 4 phyla included Firmicutes, Bacteroidetes, Proteobacteria and Actinobacteria (Supplementary Figure S3A). Actinobacteria decimated in the recipient mice (Supplementary Figure S3A). The dominant families included *Lachnospiraceae*, *Porphyromonadaceae*, *Ruminococcaceae*, *Erysipelotrichaceae*, *Enterobacteriaceae*, *Prevollaceae*, and *Bifidobacteriaceae* (Supplementary Figure S3B). Among them, *Lachnospiraceae* and *Porphyromonadaceae* were increased, and *Prevollaceae* and *Bifidobacteriaceae* were decreased in the recipient (Supplementary Figure S3B). The dominant genera included *Clostridium* XIVa, *Parabacteroides*, *Blautia*, *Gemmiger*, *Faecalibacterium*, *Bifidobacterium*, *Bacteroides, Ruminococcus*, *Escherichia*/*Shigella*, *Clostridium* XVIII, *Erysipelotrichaceae_incertae_sedis*, *Anaerostipes*, *Butyricicoccus*, *Clostridium* IV and so on (Supplementary Figure S3C).

In the donor’s gut, there were high abundance of *Faecalibacterium* (14.07%), *Prevotella* (11.31%), *Bifidobacterium* (10.69%), *Bacteroides* (8.32%), *Parabacteroides* (8.31%), *Clostridium* IV (2.87%), *Blautia* (1.22%), and *Ruminococcus* (1.04%) (Supplementary Figure S4). Nine genera were more than 1% of total bacteria in the donor man, while 12 genera in the recipient mice (Supplementary Figure S4). In these dominant genera, *Faecalibacterium*, *Prevotella*, *Bifidobacterium*, *Bacteroides*, *Dialister*, and *Clostridium* IV were decreased in the recipient’s gut, and *Clostridium* XIVa, *Parabacteroides*, *Blautia*, *Gemmiger*, *Ruminococcus*, *Escherichia/Shigella*, *Clostridium* XVIII, *Erysipelotrichaceae_incertae_sedis*, *Anaerostipes*, and *Butyricicoccus* were enriched in recipient (Supplementary Figure S4). Therefore, bacteria originating from a human intestinal microbiota selectively colonized in the gut of germ-free mice, and the dominant bacteria in the human donor were replaced by other higher abundant bacteria in recipient mice.

Statistically, the relative abundance of 1 phylum: Proteobacteria; 4 families: *Erysipelotrichaceae*, *Enterobacteriaceae*, *Clostridiaceae*, and *Peptostreptococcaceae*; and 11 genera: *Blautia*, *Escherichia/Shigella*, *Clostridium* XVIII, *Clostridium* IV, *Flavonifractor*, *Clostridium sensu stricto*, *Holdemania*, *Clostridium* XI, *Anaerotruncus*, *Clostridium* XlVb, *Dorea* were found to be significantly higher in the female recipient than the male recipient (p < 0.05, Table 1). These results suggested gut microbial colonization was sex-dependent in different taxonomic level such as phylum, family and genus.

### Comparison of overall structural base on OTU level of gut microbiota

**Figure 2:**
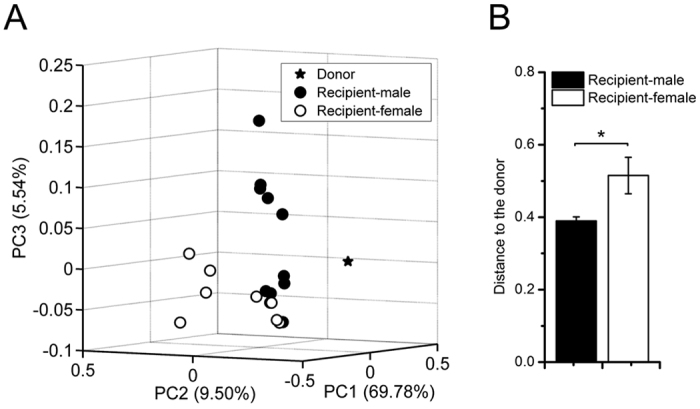
The overall structure of colonic content microbiota of male recipient mice was separated from that of female recipient, and closer to the donor. (**A**) PCoA score plot based on weighted UniFrac metrics. Each point represented the fecal microbiota of a mouse. (**B**) Weighted UniFrac distances to the donor of the two groups. Differences were assessed by Mann-Whitney test. *P < 0.05. n = 1 for Donor; n = 10 for Recipient-male group; n = 9 for Recipient-female group.

To provide an overview of the gut microbiota composition of the donor and the two recipient groups, principal coordinate analysis (PCoA) based on weighted UniFrac distances of OTU relative abundance matrix was performed. Plotted PCoA scores clearly separated the donor, the male recipient group and the female recipient group (Fig. 2A). Permutational multivariate analysis of variance (PERMANOVA) derived from weighted UniFrac distance confirmed a significant separation between the microbiota of male and female mice (p = 0.0149, Fig. 2A). Moreover, the distance to the donor were significantly lower in male recipient than female, suggesting that male recipient had more similar gut microbiota to the donor’s, compared with female recipient (Fig. 2B).

PCoA plot and PERMANOVA based on unweighted UniFrac and Bray-Curtis distances also confirmed that the overall structure of gut microbiota was significantly different between male and female groups (p < 0.05, Figure S5). However, the distances to the donor of the male and female recipient based on these algorithms were not significantly different. It was weighted UniFrac but not Euclidean/Bray-curtis/unweighted UniFrac algorithms that showed differences between the distances to the donor of male and female mice, and this might because abundance and evolutionary distance of the gut bacteria together determined the overall structure deviation from the donor.

### Comparison of gut microbiota composition at the phylotype level

**Figure 3:**
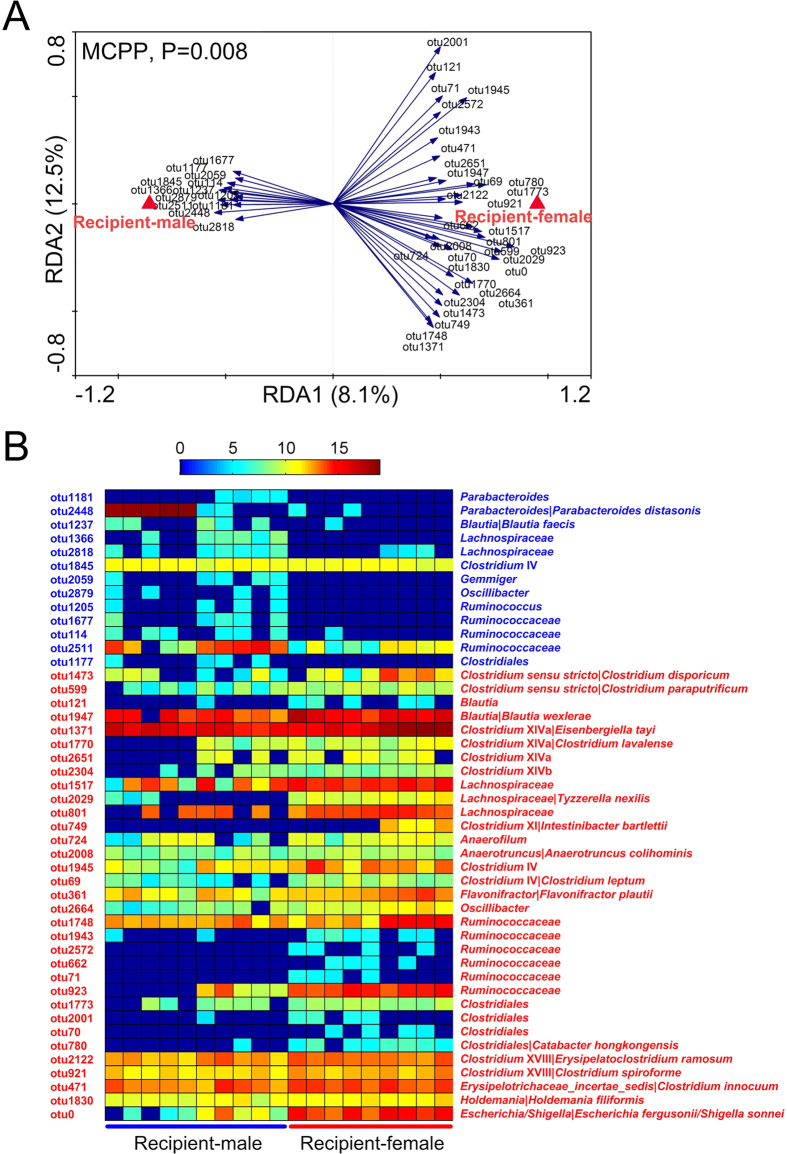
Forty-six bacterial OTUs that were different in abundance between male and female recipient according to redundancy analysis (RDA). (**A**) Biplot of the RDA between male and female recipient on relative abundance of OTUs. Constrained explanatory variables were indicated by red triangles. Blue arrows indicated the OTUs with more than 20% variability in values explained by the canonical axis. Upper left showed P-value of Monte Carlo Permutation Test. (**B**) Heatmap of the abundance of the 46 OTUs. Rows corresponded to 13 OTUs enriched in male recipient (blue), and 33 OTUs more in female recipient (red). Columns represented each mouse in the two groups. The taxonomy of the OTUs (family/genus/species) was shown on the right.

Redundancy analysis (RDA) models was employed to identify specific bacterial phylotypes for which the abundance was different between the two groups of mice (Fig. 3). Gut microbiota was significantly different between the male and female recipient (p = 0.008, Fig. 3A). Differential OTUs were revealed along the canonical axis, which explained 8.1% of the variance in OTU abundance (Fig. 3A).

There were 46 OTUs different between male and female recipient mice, with 13 OTUs higher in male mice and 33 OTUs enriched in female mice (Fig. 3B). The male-biased OTUs including the representatives from *Parabacteroides distasonis* were highly abundant in that group. The OTUs higher in abundance in the female recipient included the bacteria belonging to *Clostridium sensu stricto* (*Clostridium disporicum* and *Clostridium paraputrificum*), *Clostridum* XIVa (*Eisenbergiella tayi*, *Clostridium lavalense*), *Clostridium* XIVb, and *Clostridium* XVIII (*Erysipelatoclostridium ramousum*, *Clostridium spiroforme*), *Clostridium innocuum*, *Flavonifractor plautii* (formerly *Clostridium orbiscindens*), *Holdemania filiformis*, and *Escherichia fergusonii/Shigella sonnei* (Fig. 3B). *Blautia* and *Clostridium* IV have different behaviors among OTUs. *Blautia faecis* and otu1845 from *Clostridium* IV were higher in male recipient, while *Blautia wexlerae* and *Clostridium leptum* (belonging to *Clostridium* IV) were enriched in female recipient (Fig. 3B). Additionally, the relative abundance of some OTUs (such as otu1770, 923, 749, 71 and 2001) were more similar among the mice in the same cage than those in another cage in each gender, just as shown in the previous studies^22^,^23^,^24^. These results indicated that the gut microbiota of human donor differentially colonized in recipient mice of different sexes at the species level.

Then, a linear discriminant analysis (LDA) effect size (LEfSe) was used to confirm the above results (Supplementary Figure S6). Thirty-four bacteria OTUs were different in abundance between male and female recipient (Supplementary Figure S6B). Ten OTUs were higher in male mice, including *Parabacteroides distasonis*, *Blautia faecis* and otu1845 from *Clostridium* IV; while 24 OTUs were higher in female mice, such as *Clostridium paraputrificum*, *Clostridium lavalense*, *Clostridium leptum*, *Flavonifractor plautii*, *Holdemania filiformis*, and *Escherichia fergusonii/Shigella sonnei* (Supplementary Figure S6B). Among these OTUs, totally 21 OTUs were also identified according to RDA (Fig. 3, Supplementary Figure S6C), and in line with the taxonomy differences (Table 1), which indicated these bacterial phylotypes were indeed closely related to sex. Also, there were 13 and 25 OTUs were uniquely identified by LEfSe and RDA, respectively (Supplementary Figure S6C), because the principles and algorithms were different between the two methods^25^,^26^. Although these OTUs were divergent, they belonged to the same taxa, such as *Lachnospiraceae*, *Ruminococcaceae*, *Clostridium* XIVa, and *Clostridiales* (Supplementary Figure S6C).

### Comparison of correlation networks of differential OTUs between two sexes of recipient

**Figure 4:**
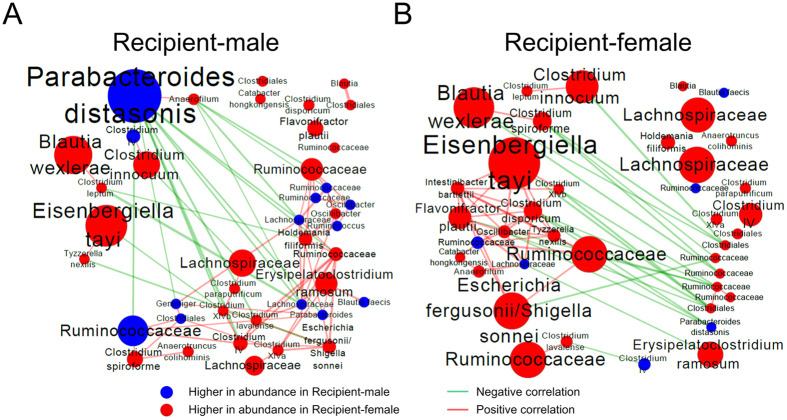
Gut microbial symbiosis and competition were distinct in male and female recipient. **(A**) Co-abundance network of differential OTUs in Recipient-male group; (**B**) Co-abundance network of differential OTUs in Recipient-female group. Blue nodes showed OTUs with higher abundance in male recipient. Red nodes showed OTUs higher in abundance in female recipient. The size of the nodes indicated OTU abundance. Connecting lines represented Spearman correlation coefficient values above 0.5 (red) or below −0.5 (green).

To identify the symbiosis and competition relationship among these sexually differential phylotypes, Spearman’s rank correlation test was employed (Fig. 4). In male recipient, a co-abundance cluster was formed among some bacteria with relatively low abundance, such as *Holdemania filiformis*, *Clostridium paraputrificum*, *Clostridium lavalense*, *Clostridium* XIVb, *Clostridium* IV, *Escherichia fergusonii/Shigella sonnei*, etc; and high abundant *Parabacteroides distasonis* in male was significantly negatively correlated with 9 OTUs, such as *Clostridium lavalense*, *Clostridium* XIVb, *Clostridium* IV, etc, which were in the co-abundance cluster (Fig. 4A). Meanwhile, the co-abundance cluster in the female recipient was composed of highly abundant OTUs including *Eisenbergiella tayi*, *Clostridium disporicum*, and *Flavonifractor plautii*. And *Parabacteroides distasonis* was significantly negatively correlated with *Clostridium disporicum*, *Rominococcaceae* and *Oscilibacter* (Fig. 4B). All above indicated that the interactions among these differential OTUs were different between the two sexes.

## Discussions

Although germ-free mice were applied broadly in studying the etiological effects of gut microbiota, the sexual disparity of human gut microbiota colonization in germ-free mice largely remains unknown. In this study, we found that female recipients had significantly different gut microbiota colonization compared to males. The differences might lie in the following four aspects: (1) Female recipients had higher bacteria diversity than male; (2) Overall structure of gut microbiota of female recipient were significantly separated from those of male recipient, and farther away from the donor’s than male recipient; (3) The differences of gut microbiota colonization between two sexes occurred at the species level; (4) The interactions among the differential OTUs were sexually distinct. Our results indicated that sex was a force shaping gut microbiota.

In this study, we compared the gut microbiota composition of male and female germ-free recipient mice after transplanting fecal bacteria from a healthy adult man. Shannon diversity analysis revealed that female recipient’s gut had bacteria with higher species diversity than that of male. In a previous study, post-pubescent female mice harbored bacteria with higher Shannon diversity index than males, which was in concert with our results^12^. Additionally, we demonstrated that it was significantly distinct between the gut microbial overall structure of male and female recipient by using unsupervised multivariate analysis. Furthermore, we performed the supervised multivariate statistical analysis to identify the species-level phylotypes causing these differences. In male recipient, the enriched species included some potentially beneficial bacteria, such as *Parabacteroides distasonis* (belonging to Bacteroidetes) and *Blautia faecis*, which was consistent with previous researches^5^,^8^,^10^,^11^. *P*. *distasonis* could reduce the severity of acute and chronic colitis in the dextran sulphate sodium-induced mice model by modulating immunity and microbiota composition^27^. *B*. *faecis*, a kind of butyrate-producing bacteria, was significantly reduced in the patients with Crohn’s disease compared to healthy individuals^28^. While, female recipient mice prefered *Clostridium* groups (including cluster sensu stricto, XIVa, XIVb, IV, and XVIII), *Flavonifractor plautii*, and *Holdemania filiformis*, of which many produced butyrate or acetate^29^,^30^,^31^,^32^,^33^,^34^. Many previous studies showed butyrate and acetate had anti-inflammatory properties^17^,^18^,^19^,^20^,^21^,^35^. However, the female-biased phylotypes also included some opportunistic pathogen, such as *Clostridium disporicum* (belonging to *Clostridium* sensu stricto), *Clostridium leptum* (belonging to *Clostridium* IV), *Clostridium spiroforme* (belonging to *Clostridium* XVIII), *Clostridium innocuum*, and *Escherichia fergusonii/Shigella sonnei*, which were implicated in the development of infection or colitis^36^,^37^,^38^,^39^,^40^,^41^,^42^. Moreover, the interactions among these bacteria were different as well, suggesting that the gut of male and female mice have ecologically distinct environments.

Hereinabove, male and female recipients were colonized by different bacteria, which showed different or even opposite effects on diseases. This fact might account for why male and female individuals reacted differently to the same disease. So fecal microbial transplantation to germ-free mice from more donors should be carried out to verify the above results in the future. In that case, it is recommended to take a comprehensive picture of relationship between gut microbiota and disease through utility of both sexes of germ-free mice model.

Although female recipients had more variety of bacteria than males, especially more butyrate/acetate-producing bacteria and opportunistic pathogen, the overall structure of their gut microbiota was farther away from that of the donor than male recipient. The overgrowth of some bacteria in female mice’s gut might lead to the more significant deviation of the whole gut microbiota from the male donor than male recipient. It might reflect that the gut microbiota colonized in the mice of the same gender was more similar to that from the donor. In order to fully illustrate this point, it would be necessary to compare the colonization pattern of gut microbiota from female donor in two sexes of mice.

Several mechanisms might involve in sex modulation of gut microbiota colonization. Host sex hormones can modulate microbiota composition^5^,^12^. Sex differences in gut microbiota did not appear before puberty, and removal of the androgen source by castration would drive male microbiota closer to female microbiota, which suggested that sexual maturation and sexual hormones were major determinants of the gut microbial community structure difference between two genders^5^,^12^. In addition, immune function differed between sex, with more macrophage numbers and inflammatory cytokines secretion in male, influenced gut microbiota composition^5^. Our study did not clarify the mechanisms driving sexual differentiation of gut microbiota colonization at present. Instead, we demonstrated for the first time that sex did control microbiota colonization pattern in adult germ-free mice.

In brief, our results represented an example of microbiota colonization depending on sexes of germ-free mice. Female recipient mice processed higher bacteria diversity, unique overall structure and individual phylotype pattern, and different bacteria interactions in comparison to the male recipient. These findings suggested that the sex of gnotobiotic mice should be taken into account when investigating modulatory effects of gut microbiota on diseases, and it’s better to tailor the host sex in the treatment of diseases through “gut microbiota-targeted” strategies according to the bacterial colonization ability.

## Materials and Methods

### Animal experiments

The donor was a 25-year-old healthy man, without medication for at least 6 months. Before donating the stool, he had 7 days of vegetarian diet and inulin intake, in order to increase the abundance of potential beneficial bacteria^43^,^44^,^45^. Written informed consent was obtained from this donor. Fresh stool was collected and diluted 5-fold and homogenized in sterile pre-reduced 0.01 M phosphate buffer saline (pH 7.4). Food derived debris of too large size was removed through sterile gauze.

Ten male and ten female germ-free C57BL/6J mice (6–8 weeks old) were purchased from Shanghai Laboratory Animal Center (SLAC), Chinese Academy of Sciences, Shanghai, China. They were fed on normal chow diet provided by SLAC. After two weeks’ acclimatization, the 20 mice were divided into 2 groups: male group and female group. The ten mice in each group were housed in two cages (five per cages). The human fecal suspension was administered orally by gavage to the germ-free mice at a dosage of 100 ul/mice once daily in the first two days. The trial lasted for 7 days. One female mouse died after inoculation. At the end of the trial, colonic contents of each mouse were collected, and frozen in liquid nitrogen immediately for further analysis.

The use of human fecal sample was approved by Medical Ethics Committee of Shanghai General Hospital (No. 2016KY072), and all experiments were performed in accordance with the relevant guidelines and regulations. All the animal experiments were carried out in strict accordance with the Guidelines for Care and Use of Laboratory Animals of SLAC. The protocol was approved by the Institutional Animal Care and Use Committee of SLAC (SLAC-20160320001).

### Sequencing of gut bacterial 16S rRNA gene V3-V4 region on the Illumina MiSeq platform

Genomic DNA was extracted from colonic content samples collected from two groups of mice using bead beating and an InviMag^®^ DNA kit (Invitek, Berlin, Germany) method as reported previously^46^. The extracted DNA from each sample was used as the template to amplify the V3-V4 region of the 16S rRNA gene. The bacterial universal primer pair in the V3-V4 region consisted of the forward primer 5′-TCGTCGGCAGC GTCAGATGTGTATAAGAGACAGCCTACGGGNGGCWGCAG-3′and the reverse primer 5′-GTCTCGTGGGCTCGGAGATGTGTATAAGAGACAGGACTACHVGG GTATCTAATCC-3′. A subsequent limited-cycle amplification step was performed to add multiplexing indices and Illumina sequencing adapters. Libraries were normalized and pooled, and sequenced on the MiSeq platform according to the manufacturer’s instructions (Illumina, CA, USA).

### Bioinformatics and statistical analysis of sequencing data

All raw reads were sorted into different samples according to the multiplexing indices. High-quality sequences were selected under the following criteria by using Usearch 7.0.1090: 1) sequences at both ends should be cut at the nucleic acid bases whose quality was lower than Q2; 2) the overlap of splicing sequences should be longer than 50 bp, and the length of complete sequences should be longer than 400 bp, and 3) the expected error of the complete sequences should be less than 1 base. PCR primers were truncated out afterwards.

Bioinformatics analysis of sequencing data was performed using QIIME (v1.8.0)^47^. All high-quality sequences were aligned against Greengenes core dataset with by PyNAST multialigner, and percent identity ≥75% with the reference sequences were regarded as bacterial sequences and used for further analysis. Operational taxonomic units (OTU) delineation was performed with UCLUST at 97% similarity level. Chimera sequences identified by Uchime and singletons were discarded. Bacterial diversity was assessed with the Shannon Diversity Index and rarefaction analysis based on abundance of OTU sequences. Principal coordinate analysis (PCoA) was performed on unweighted and weighted UniFrac, Bray-Curtis and Euclidean distances of the relative abundances (normalized for each sample) of OTUs to visualize whether there was segregation of gut microbiota structure between two animal groups or not. The statistical significance of PCoA plots of two groups was assessed by permutational multivariate analysis of variance (PERMANOVA) test in MATLAB R2010a (The MathWorks, Inc., Natick, MA, USA).

To identify OTUs separating the two groups, redundancy analysis (RDA) models and linear discriminant analysis effect size (LEfSe) were contrasted based on the relative abundance of OTUs. RDA was performed with Canoco for Windows 4.5 (Microcomputer Power, NY, USA) according to the manufacturer’s instructions. Statistical significance was assessed by Monte Carlo Permutation Procedure (MCPP) with 499 random permutations under the full model. In LEfSe, OTUs were picked out when alpha value of the factorial Kruskal–Wallis test was <0.05, and the logarithmic LDA score is >2.0: (http://huttenhower.sph.harvard.edu/galaxy).

Taxonomical assignments of representative sequences were determined using RDP classifier with a bootstrap cutoff of 80%. Species-level classifications were ascertained by using MegaBLAST with a greater than 99% similarity to a reference sequence derived from a cultured isolate, and no similarity at greater than 97% to any other cultured species^44^. The significance of differences in taxon-level between the two groups was tested by an embedded script in QIIME (group_significance.py). Differences were considered significant when FDR < 0.20.

The symbiosis and competition relationships between the differential OTUs were evaluated by Spearman’s rank correlation based on the relative abundances of OTUs. The network in each group was visualized by using Cytoscape v3.1.1^48^.

## Additional Information

**Accession codes:** The sequence information in this paper has been submitted to the GenBank Sequence Read Archive database under accession number SRP076247. 

**How to cite this article**: Wang, J.-j. *et al*. Sex differences in colonization of gut microbiota from a man with short-term vegetarian and inulin-supplemented diet in germ-free mice. *Sci. Rep.* **6**, 36137; doi: 10.1038/srep36137 (2016).

**Publisher’s note:** Springer Nature remains neutral with regard to jurisdictional claims in published maps and institutional affiliations.

## Supplementary Material

Supplementary Information

## Figures and Tables

**Table 1:** List of taxa that were significantly different between male and female recipient.

Taxon	P value	FDR	Relative abundance (%)		Fold
			Recipient-male	Recipient-female	
**Phylum**					
Proteobacteria	0.0004	0.0022	0.18 ± 0.12	2.31 ± 0.43	12.8
**Family**					
*Erysipelotrichaceae*	0.0090	0.0868	2.05 ± 0.24	3.17 ± 0.23	1.60
*Enterobacteriaceae*	0.0004	0.0129	0.18 ± 0.12	2.31 ± 0.43	12.8
*Clostridiaceae*	0.0015	0.0210	0.06 ± 0.02	0.50 ± 0.17	8.30
*Peptostreptococcaceae*	0.0135	0.0976	0.01 ± 0.00	0.17 ± 0.07	17.0
**Genus**					
*Blautia*	0.0071	0.0693	2.27 ± 0.49	6.79 ± 1.57	3.00
*Escherichia/Shigella*	0.0004	0.0262	0.18 ± 0.12	2.30 ± 0.43	12.8
*Clostridium* XVIII	0.0071	0.0693	0.97 ± 0.11	1.51 ± 0.13	1.60
*Clostridium* IV	0.0019	0.0377	0.49 ± 0.05	1.15 ± 0.21	2.30
*Flavonifractor*	0.0338	0.1532	0.41 ± 0.06	0.83 ± 0.18	2.00
*Clostridium sensu stricto*	0.0015	0.0377	0.06 ± 0.02	0.50 ± 0.17	8.30
*Holdemania*	0.0055	0.0693	0.15 ± 0.02	0.25 ± 0.02	1.70
*Clostridium* XI	0.0135	0.0993	0.01 ± 0.00	0.17 ± 0.07	17.0
*Anaerotruncus*	0.0338	0.1532	0.03 ± 0.01	0.06 ± 0.01	2.00
*Clostridium* XlVb	0.0322	0.1532	0.02 ± 0.01	0.04 ± 0.01	2.00
*Dorea*	0.0085	0.0713	0.00 ± 0.00	0.03 ± 0.02	—

Note: Data were shown as means ± SEM. Differences were assessed in QIIME.
