# Supplementary material for: Sex differences in colonization of gut microbiota from a man with short-term vegetarian and inulin-supplemented diet in germ-free mice
Source: Sci Rep. 2016 Oct 31;6:36137. doi: 10.1038/srep36137 (PMC5086848; doi:10.1038/srep36137)
Supplement: Supplementary Information [file srep36137-s1.pdf]

Sex differences in colonization of gut microbiota from a man with short-term vegetarian and inulin-supplemented diet in germ-free mice

Jingjing Wang, Jing Wang, Xiaoyan Pang, Liping Zhao, Ling Tian, Xingpeng Wang

Supplementary Information

Summary

The supplementary information includes five supplementary figures and supplementary discussions.

Supplementary Figures
Figure S1

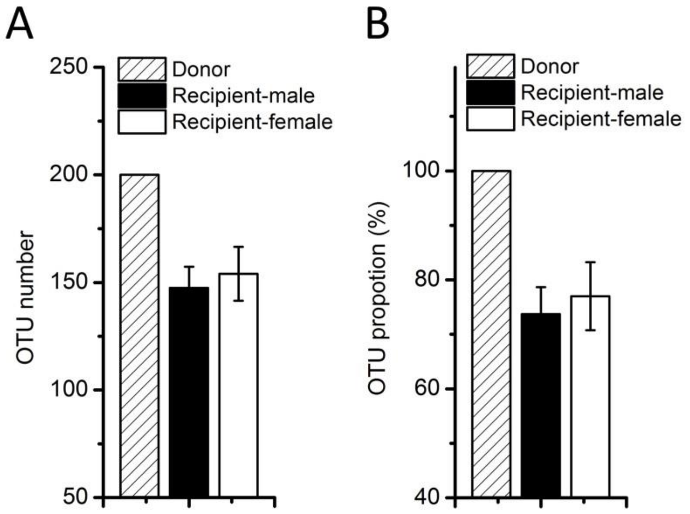

Figure S1 Gut microbiota colonizing in the gut of recipient mice. (A) The number of OTUs colonizing in the gut of donor, male recipient and female recipient, respectively; (B) The percentage of OTUs colonizing in recipients relative to OTUs in donor. Data were shown as means ±SEM. n=1 for Donor; n=10 for Recipient-male group; n=9 for Recipient-female group.

Figure S2

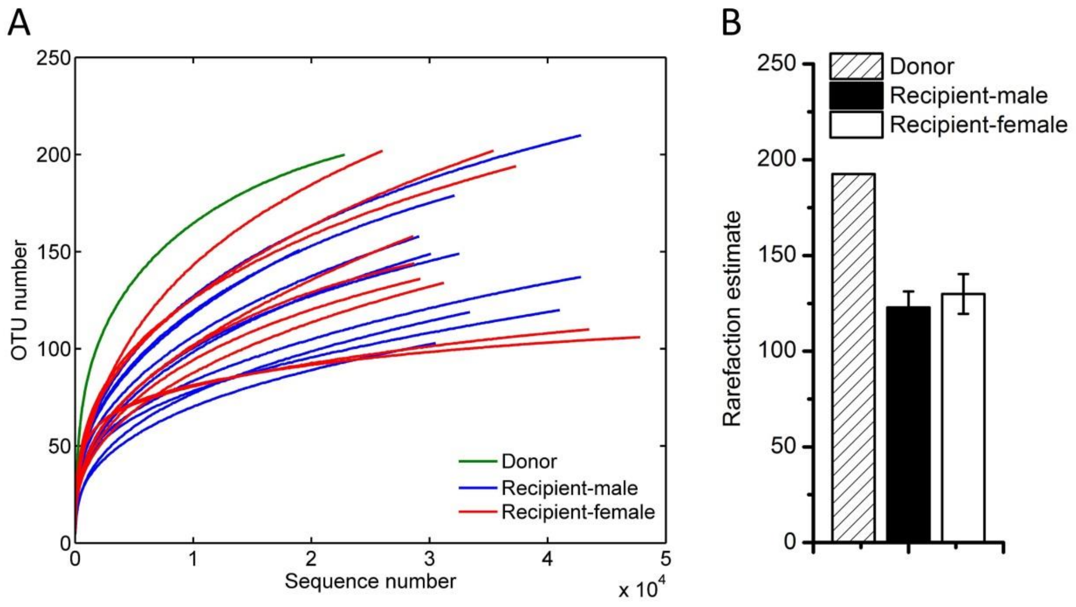

Figure S2 Rarefaction curves and Rarefaction estimate of each group. Data were shown as means ± SEM. n=1 for Donor; n=10 for Recipient-male group; n=9 for Recipient-female group.

Figure S3

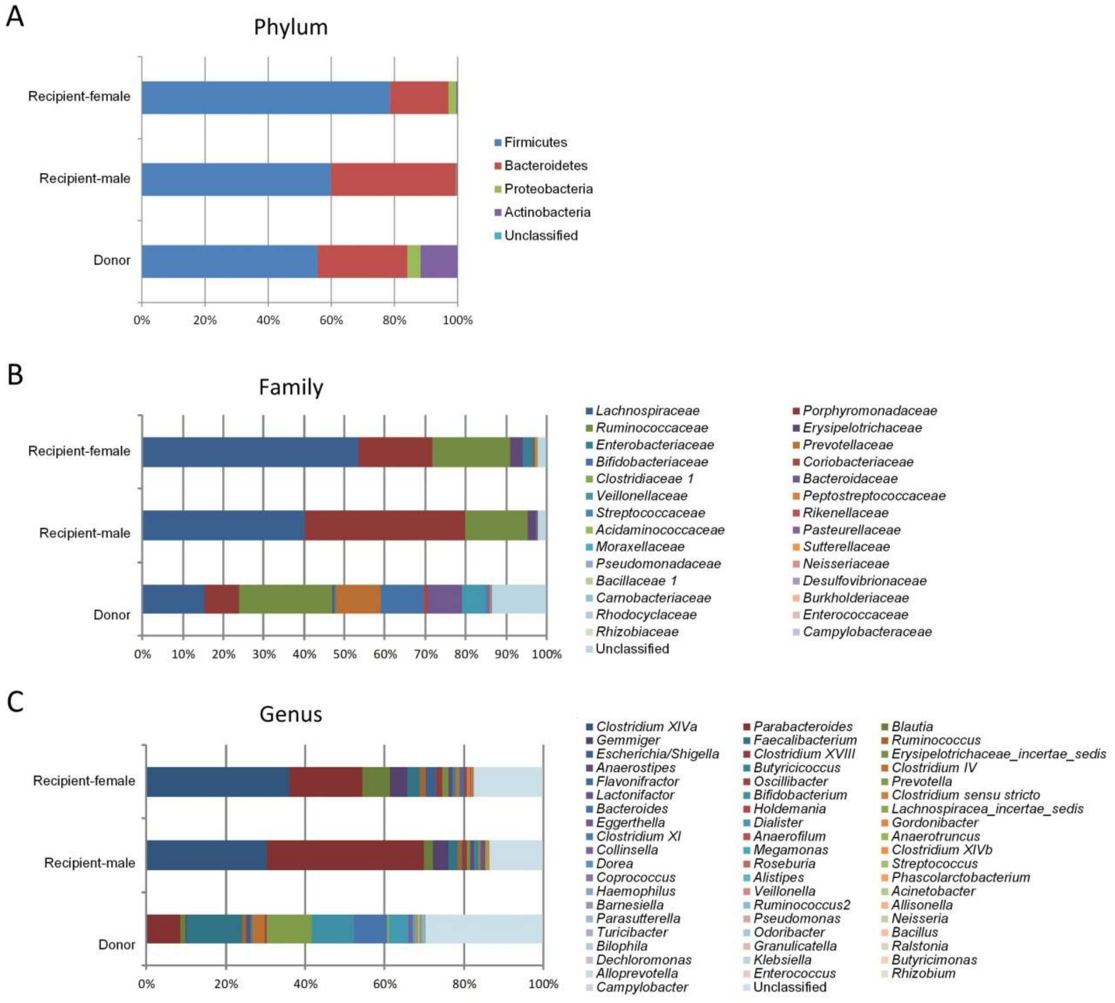

Figure S3 Gut microbial composition of donor, male and female recipient in phylum (A), family (B) and genus (C) levels.

Figure S4

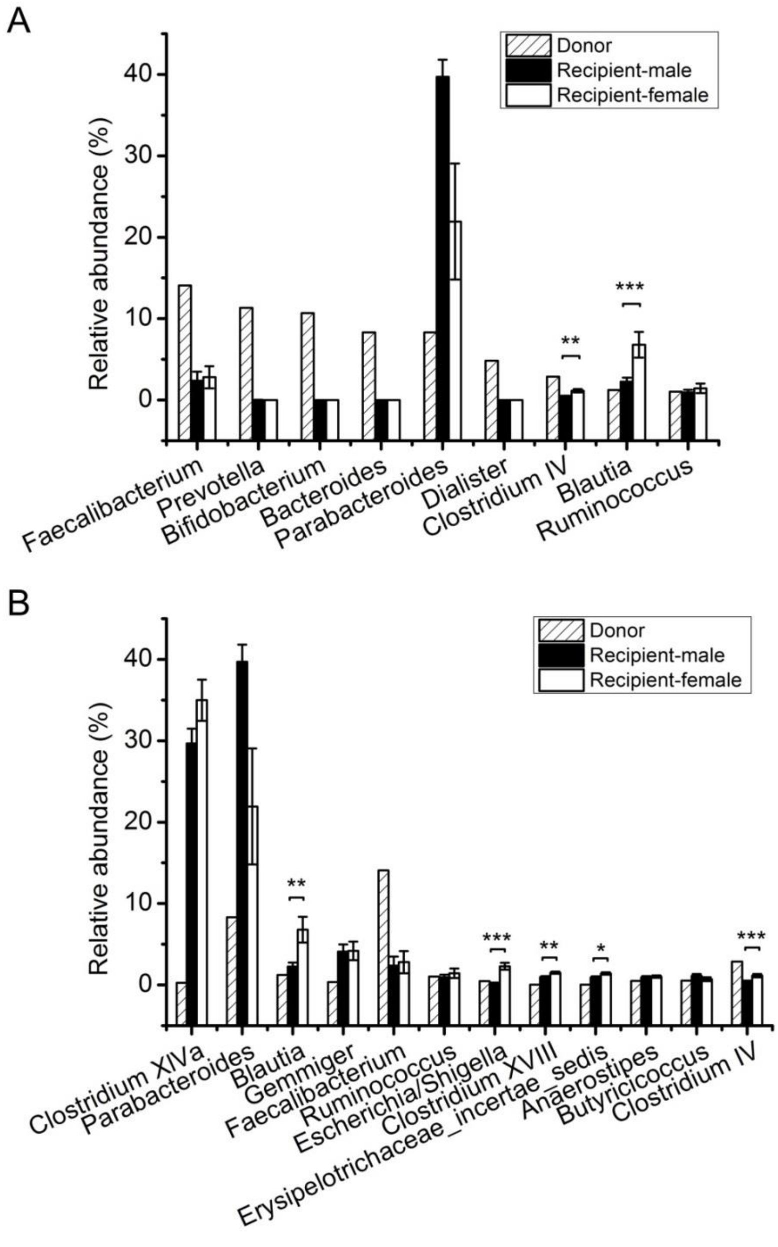

Figure S4 Relative abundance of predominant genera (> 1% in donor or recipient). (A) Relative abundance of predominant genera (> 1% in donor); (B) Relative abundance of predominant genera (> 1% in male or female recipients). Data were shown as means ± SEM. Differences were assessed by Mann-Whitney test. * $P < 0.05$, ** $P < 0.01$, *** $P < 0.001$. N=1 for Donor; n=10 for Recipient-male group; n=9 for Recipient-female group.

Figure S5

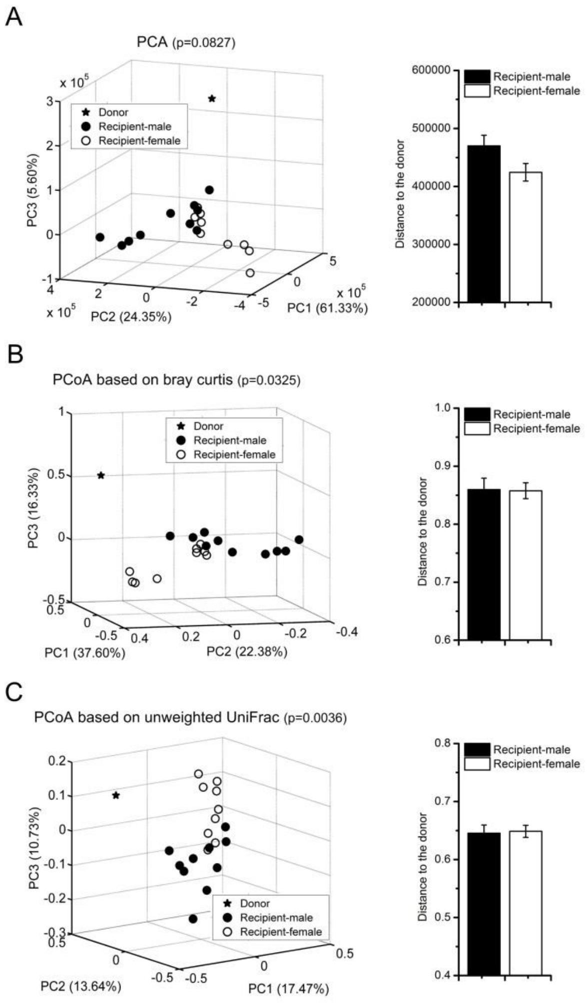

Figure S5 The overall structure of colonic content microbiota were separated between male and female recipient. (A) PCA score plot and Euclidean distance to the donor of the two groups. (B) PCoA score plot based on Bray-curtis metrics and Bray-curtis distances to the donor of the two groups. (C) PCoA score plot based on unweighted UniFrac metrics and unweighted UniFrac distances to the donor of the two groups. Each point represented the fecal microbiota of a mouse. P value was calculated by PERMANOVA between male and female recipient mice. n=1 for donor; n=10 for Recipient-male group; n=9 for Recipient-female group.

Figure S6

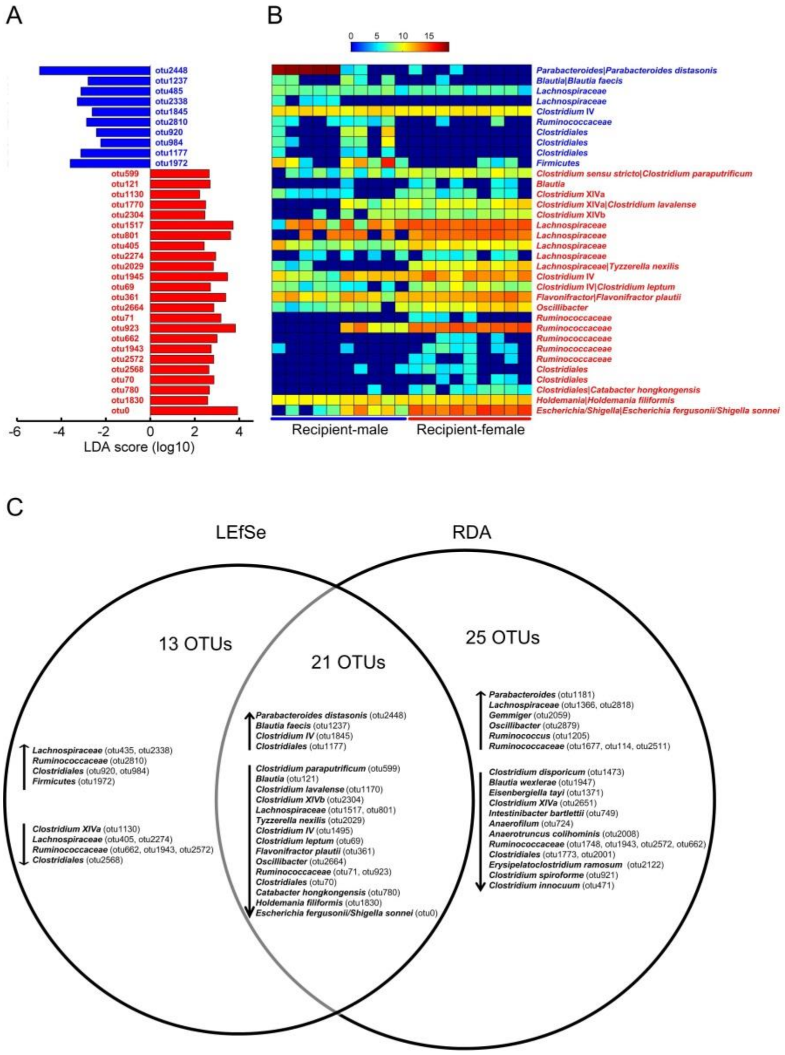

Figure S6 Thirty-four bacterial OTUs that were different in abundance between male and female recipient according to Linear discriminant analysis Effect Size (LEfSe). (A) LDA scores computed for OTUs differentially abundant between male and female recipient groups. Ten OTUs were higher in male recipient (blue), and twenty-four OTUs were higher in female recipient (red). (B) Heatmap which showed the relative abundance of altered OTUs. Columns represented each mouse in the two groups. The taxonomy of the OTUs (family/genus/species) was shown on the right. (C) Venn diagrams of OTUs identified by RDA and/or LEfSe. ↑ means increased OTUs in male than female, and ↓ represents decreased OTUs in male than female.

Supplementary Discussions

In our study, there were 200 OTUs in the donor's gut; while, in Yatsunenko's work (Reference 9), there are about 900-1500 in the adults' gut. We can explain the discrepancy by the following aspects:

1) Different methods of sequencing and data analysis

We used Illumina MiSeq platform to sequence the fecal bacteria samples, and got 24,134 reads from the donor's sample, much less than Yatsunenko's study (1,803,250 ±562,877 reads/sample). This might induce the reduction of OTU number.

After getting 2934 OTUs, we removed the chimeras and singletons, and finally got 554 OTUs. Yatsunenko and his colleagues used OTUs with abundances >0.1%. The different standards of data analysis also might induce the changes of OTUs number.

2) Different source of fecal samples

Our donor comes from China, and in Yatsunenko's job, the cohort are from Amazonas of Venezuela, rural Malawi and US metropolitan areas. There are many differences in geography, weather, and diet. Therefore, the gut microbiota of our donor and his cohort are distinct.

Moreover, our donor had a 7-day of vegetarian and inulin-supplemented diet before donating the stool. This kind of simple diet gave the environmental pressure to the growth of gut microbiota, and reduced the species number (OTU number) of gut microbiota.
